# Palifermin for the protection and regeneration of epithelial tissues following injury: new findings in basic research and pre-clinical models

**DOI:** 10.1111/jcmm.12091

**Published:** 2013-09-24

**Authors:** Paul W Finch, Lawrence J Mark Cross, Daniel F McAuley, Catherine L Farrell

**Affiliations:** a Croton-on-Hudson, NY, USA; b Centre for Infection and Immunity, The Queen's University of BelfastBelfast, N Ireland, UK; c Novel Therapies Division, Epistem Ltd.Manchester, UK

**Keywords:** palifermin, KGF, FGFR2b, cytoprotection, epithelial repair, GVHD, immune reconstitution, mucositis, pericardial adhesions, pre-synaptic organizing molecules, pulmonary injury, regeneration, urothelium, wound healing

## Abstract

Keratinocyte growth factor (KGF) is a paracrine-acting epithelial mitogen produced by cells of mesenchymal origin, that plays an important role in protecting and repairing epithelial tissues. Pre-clinical data initially demonstrated that a recombinant truncated KGF (palifermin) could reduce gastrointestinal injury and mortality resulting from a variety of toxic exposures. Furthermore, the use of palifermin in patients with hematological malignancies reduced the incidence and duration of severe oral mucositis experienced after intensive chemoradiotherapy. Based upon these findings, as well as the observation that KGF receptors are expressed in many, if not all, epithelial tissues, pre-clinical studies have been conducted to determine the efficacy of palifermin in protecting different epithelial tissues from toxic injury in an attempt to model various clinical situations in which it might prove to be of benefit in limiting tissue damage. In this article, we review these studies to provide the pre-clinical background for clinical trials that are described in the accompanying article and the rationale for additional clinical applications of palifermin.

- Introduction
- Updates of KGF basic research
  – Control of epithelial cell-specific FGFR2b expression
  – KGF/FGFR2b signalling mechanisms
  – FGFR2b signalling in tumourigenesis and tumour suppression
  – KGF family members as pre-synaptic organizing molecules
- Pre-clinical models of KGF protective and regenerative effects
  – Palliative care in the oncology setting
  – Intestinal mucosal injury
  – Graft-versus-host disease (GVHD) and immune reconstitution
  – Pulmonary disease
  – Dermatological applications
  – Urothelium
  – Pericardium
- Conclusions and perspectives

## Introduction

The fibroblast growth factors (FGFs) comprise a structurally related family of 22 molecules that can be grouped into seven subfamilies on the basis of sequence similarities, conserved chromosomal locations and functional properties [1]. Most FGFs are secreted proteins which act as autocrine or paracrine factors by binding high affinity receptor tyrosine kinase molecules (FGFR1 – 4) in the presence of heparan sulphate (HS) glycosaminoglycans. This leads to the formation of FGF-FGFR-HS dimers in which transphosphorylation of the cytoplasmic kinase domains activates various signal transduction cascades that have diverse roles in regulating cell proliferation, migration, and differentiation during vertebrate development, as well as homeostasis, response to injury and tissue repair in adult animals [2–4]. Alternative RNA splicing results in two different versions of an external Ig-like domain III (referred to as domains IIIb and IIIc) in FGFRs 1 – 3, with the IIIb isoform restricted to cells of epithelial lineage, whereas the IIIc isoform is preferentially expressed by mesenchymal lineages [5–7]. In addition, FGFs 15, 19, 21 and 23 regulate metabolism at postnatal stages *via* an exocrine mechanism by virtue of reduced heparin-binding affinity, although still in an FGFR-dependent manner [8]. Alternatively, FGFs 11-14 regulate the electrical excitability of neurons by an intracrine FGFR-independent mechanism [9, 10].

Keratinocyte growth factor (KGF), which was first isolated as an epithelial mitogen from the conditioned medium of human embryonic lung fibroblasts, was the seventh member of the FGF family to be identified, and so is also known as FGF7 [11, 12]. Phylogenetic and gene location analysis indicates that KGF, along with FGF10 and FGF22 comprise an FGF subfamily. Keratinocyte growth factor and FGF10 are primarily produced by cells of mesenchymal origin [11–14] whereas FGF22 is expressed throughout the epidermis and hair follicle keratinocytes in skin [15, 16], as well as by cerebellar granule cells during the period when they receive synapses [17]. Keratinocyte growth factor, FGF10 and FGF22 all strongly activate FGFR2b, [18, 19], whereas FGF10 and FGF22 also show weak activity towards FGFR1b. No other activity towards any other FGFR isoforms has been detected [19]. The restricted pattern of FGFR2b expression primarily by epithelial cells and the high specificity of FGF7 and FGF10 for this FGFR isoform support the hypothesis that they function as paracrine signals that mediate mesenchymal-epithelial communication [20]. In contrast, the expression pattern of FGF22 suggests that it may function as a presynaptic organizing molecule in the developing brain, as well as in cutaneous development and repair. Disruption of FGF10 results in severe defects in the development of organs requiring branching morphogenesis, such as the lungs, pancreas and salivary glands [13, 21] indicating a critical role in embryonic development. However, KGF knockout mice are viable [22], and essentially normal, albeit with minor alterations in hair characteristics [22], kidney development [23] and urothelial stratification in the bladder [24], indicating that KGF does not play a critical role in developmental organogenesis. Rather, the up-regulation of KGF expression in adult animals following injury to a number of different tissues [25–31] as well as its chronic elevation in various human inflammatory diseases [32–35], suggested that it participates in a wide variety of epithelial preservation and/or repair processes. Both *in vitro* and *in vivo* studies have demonstrated that KGF has potent cytoprotective and regenerative effects on epithelial tissues subjected to a variety of toxic exposures [20, 36–40]. In a model of inflammatory bowel disease (IBD) induced by treatment with dextran sulphate, KGF knockout mice exhibited more severe colonic inflammation and a delay in tissue repair compared with wild-type mice [41], implying a specific, non-redundant role in limiting damage to the intestine. These beneficial effects arise from multiple mechanisms that act collectively to strengthen the integrity of the epithelium by stimulating cell proliferation, migration, differentiation, survival, DNA repair, and induction of enzymes involved in the detoxification of reactive oxygen species [20].

For the reasons outlined above, KGF has been the subject of intensive efforts to identify clinical applications in which the integrity of epithelial surfaces is at risk, and the preservation or rapid restoration of these tissues would be of benefit. Currently, a truncated form of recombinant KGF (palifermin, brand name Kepivance) has been approved for the treatment of severe oral mucositis in patients with hematologic malignancies who have been treated with high doses of chemotherapy and radiation prior to autologous blood progenitor cell transplantation [42]. The purpose of this article is to briefly summarize recent developments in KGF basic research, and then review the preclinical studies of KGF action that have either provided the foundation for a series of ongoing clinical trials relating to the management of mucositis resulting from cancer treatment regimens, or the impetus for clinical trials relating to new indications, such as the enhancement of chronic and acute wound healing, the control of graft-versus-host-disease (GVHD) and immune reconstitution following allogeneic stem cell transplantation, or containment of lung injury following toxic exposure.

## Updates of KGF basic research

### Control of epithelial cell-specific expression of FGFR2b

The exquisite specificity of KGF for cells of epithelial origin is determined by alternative splicing of the *FGFR2* gene, in which exons IIIb and IIIc undergo exclusive and tissue-specific alternative splicing. In this section, we review the mechanisms that act to coordinate the epithelial cell-specific splicing programme.

As described above, the FGFR2b splice variant, which KGF binds to exclusively [19], is only expressed by epithelial cells, whereas FGFR2c expression is mesenchymal. Mutually exclusive use of exons IIIb or IIIc is regulated by the polypyrimidine tract-binding protein (PTB) which binds to silencing elements around exon IIIb, resulting in its repression [43, 44]. In addition, different histone modification signatures have been demonstrated in the intron separating the IIIb and IIIc exons, modulation of which can result in splice site switching [45]. Specifically, H3-K36me3 and H3-K4me1 were enriched in IIIc expressing cells, whereas H3-K27me3, H3-K4me3, and H3-K9me1 were reduced in cells expressing transcripts containing IIIb. Overexpression of the H3-K36 methyltransferase, SET2, significantly reduced the inclusion of the IIIb isoform, but did not affect IIIc expression [45]. In the case of *FGFR2* gene expression, histone marks affect splicing outcome by influencing the recruitment of an adaptor system consisting of H3-K36me3, its binding protein MRG15, and the splicing regulator PTB [45].

Two paralogous epithelial cell-specific RNA binding proteins, ESRP1 and ESRP2, have been identified as essential regulators of FGFR2 splicing [46]. Ectopic expression of either protein in cells expressing FGFR2c caused a switch in endogenous splicing to the epithelial isoform. Conversely, knockdown of both factors in cells that express FGFR2b results in a switch to expression of the IIIc isoform [46]. ESRP1 and ESRP2 positively and negatively regulate diverse alternative splicing events in a epithelial-specific manner [47], and abrogation of the global ESRP-regulated phenotype induces phenotypic changes in cell morphology associated with epithelial-mesenchymal transition [48]. Thus, control of alternative splicing of the FGFR2 gene, in which exons IIIb and IIIc undergo mutually exclusive and tissue specific alternative splicing, is complex and involves the recruitment of several different splicing regulators mediated by specific chromatin binding proteins, and alternative sets of histone modification.

### KGF/FGFR2b mediated signalling mechanisms

A more complete understanding of the signalling mechanisms utilized by KGF to elicit its cytoprotective effects on target epithelial cells will enhance efforts to maximize its pharmaceutical potency, as well as open up the possibility of beneficial combinatorial therapeutic regimens with other pharmaceuticals. In this regard, this section provides an overview of KGF mediated signalling mechanisms.

By transfecting KGF unresponsive cells with an inducible *FGFR2b* expression system, Luo and colleagues developed a model system to examine phosphotyrosine targets of KGF signalling [49]. Following KGF stimulation, tyrosine phosphorylation of multiple targets involved in growth stimulation were identified, including the multi-substrate organizer FRS2α, which mediates signalling through FGFRs [50] as well as IRS4, implicated in mediating the stimulatory effects of insulin, IGF-1, and growth hormone [51]. Phosphorylation was also observed of the canonical ERK2, a central mediator of the RAS-RAF-MAPK pathway involved in both physiological and constitutive growth [52], as well as the tyrosine phosphatase SHP2, whose activation through tyrosine phosphorylation enhances this MAPK pathway [52, 53]. Consistent with these results, transient activation of MAPK by KGF has been documented in a variety of epithelial cells [54–59]. Several proteins associated with phospholipid/intracellular calcium signalling, cell adhesion, migration and the tumourigenic phenotype, including phosphatidylinositol 3-kinase (PI-3K), were not tyrosine phosphorylated following KGF stimulation [49]. Nonetheless, PI-3K has been implicated in KGF mediated induction of lipogenic genes in pulmonary epithelial cells [60], KGF-induced differentiation of pancreatic duct cells [58], and KGF inhibition of Fas-mediated apoptosis in lung epithelial cells [61], suggesting limitations of the model assay system. Several proteins were also identified with defined roles in the attenuation of cell proliferation, including the cyclin-dependent kinase CDK2, which is inhibited by tyrosine phosphorylation after KGF treatment [62], and the tyrosine phosphatase PTPN18, which may down-regulate growth stimulatory signalling, including that elicited through ERBB family receptors [63, 64]. Finally, several protein substrates were identified with roles in maintaining nuclear structure and nuclear interactions with cytosolic components, transcription regulation, protein folding and other cellular fine structures. Many of these proteins were associated with growth inhibition and tumour suppression, including lamina-associated polypeptide 2 [65, 66], and SAP102, a member of the Dlg family of tumour suppressor proteins [67, 68]. Thus, KGF signalling through FGFR2b activates a subset of targets that generally promote cell growth, but also other novel FGFR2b-specific targets that are associated with the attenuation of cell growth and promotion of cell differentiation [49].

Keratinocyte growth factor induced the antiapoptotic Akt pathway in a mouse model of oxidant-induced lung injury, and inhibition of this activation blocked KGF-mediated protection of the lung epithelium [69]. FGFR2b interacts with the p21-activated protein kinase 4 (PAK4), a member of the PAK family of proteins that are regulated by the Rho family GTPases Rac and Cdc42. A dominant-negative PAK4 mutant blocked KGF inhibition of caspase-3-dependent apoptosis in epithelial cells subjected to oxidant stress [70]. Thus, Akt and PAK4 appear to be important mediators of KGF antiapoptotic activity. Keratinocyte growth factor activated nuclear factor κB (NF-κB) in human pancreatic ductal epithelial cells, which in turn induced expression of VEGF, matrix metalloproteinase protein-9 (MMP9) and urokinase-type plasminogen activator, and increased migration and invasion [71]. Keratinocyte growth factor also stimulated the expression of the CCAAT/enhancer binding proteins C/EBPα and C/EBPδ in lung epithelial cells and keratinocytes, which may in part mediate its effects on proliferation, migration, and lipogenesis [60, 72]. Various KGF target genes have been identified (Table 1), including the Nrf2 transcription factor [73], which presumably mediate KGF effects on proliferation, migration, differentiation, survival, DNA repair and detoxification of reactive oxygen species in target cells.

**Table 1:** Summary of genes known to be up-regulated by KGF, which have putative roles in tissue protection and regeneration

Gene encoding	Cellular process	Specific function	References
Neu differention factor/heregulin	Proliferation	Development and maintenance of several organ systems including the nervous system, heart, neuromuscular junction and breast	[283]
Mrp3 mitogen-related protein	Proliferation	Wound healing/hair follicle proliferation	[284]
Caveolin-1 and -2	Proliferation	Regulation of various signal transduction pathways	[285]
Hyaluronan Syynthase-2 and-3	Migration	Synthesis of hyaluronan, a glycosaminoglycan that enhances cell migration	[286]
Integrin α_5_	Migration	Fibronection receptor, highly upregulated following epidermal injury	[72]
Nuclear factor κβ	Migration, Angiogenesis	Transcription factor with multiple roles in growth, differentiation, apoptosis and inflammation	[71]
VEGF	Angiogenesis	Stimulation of vasculogenesis and angiogenesis	[287]
Oestrogen-responsive B box protein	Differentiation	Expression in normal epidermis but down regulation hyperproliferative epithelium suggests role in maintaining keratinocyte differentiation	[288]
Akt	Survival	Inhibition of apoptosis	[69]
P21-activated protein kinase 4	Survival	Inhibition of apoptosis	[70]
Ribosomal S6 kinase	Survival	Mediates KGF-induced Akt activation	[289]
Adenylosuccinate synthetase	Nucleotide Biosynthesis	Conversion of inosine monophosphate into adenylosuccinate	[290]
Adenylosuccinate lyase	Nucleotide Biosynthesis	*De novo* purine synthesis	[290]
Phosphoribosyl pyrophosphate synthetase	Nucleotide Biosynthesis	Generates phosphoribosyl pyrophosphate, required for *de novo* production of purine and pyrimidine nucleotides	[290]
Amidophosphoribosyl transferase	Nucleotide Biosynthesis	Key regulatory enzyme in purine biosynthesis	[290]
Hypoxanthine guanine phosphoribosyl transferase	Nucleotide Biosynthesis	Involved in the salvage pathway to recover bases and nucleosides formed during degradation of RNA and DNA	[290]
Carbamoylphosphate synthetase II	Nucleotide Biosynthesis	Catalysis of initial steps in *de novo* pyrimidine synthesis	[290]
Aspartate transcarbamylase	Nucleotide Biosynthesis	Catalysis of initial steps in *de novo* pyrimidine synthesis	[290]
Dihydroorotase	Nucleotide Biosynthesis	Catalysis of initial steps in *de novo* pyrimidine synthesis	[290]
NM23	Nucleotide Biosynthesis	Maintenance of intracellular nucleotide levels	[291]
Collagenase (MMP-1)	Tissue Remodelling	Cleavage of interstitial fibrillar collagens (types I, II, and III)	[292]
Gelatinase (MMP-9)	Tissue Remodelling	Hydrolysis of gelatin into small peptides	[293]
Stromelysin-2 (MMP-10)	Tissue Remodelling	Broad substrate specificity MMP	[294]
Collagenase-3 (MMP-13)	Tissue Remodelling	Wide selection of substrates including fibrillar collagens and other matrix and non-matrix components	[295]
Urokinase-type plasminogen activity	Tissue remodelling	Cleavage of plasminogen, the inactive form of the serine protease plasmin	[296], [293], [297]
CHL-1	Cell Cycle Progression and DNA Repair	ATP-binding protein with DNA helicase activity	[298]
Glutathione peroxidase	Detoxification of Reactive Oxygen Species	Reduction in lipid hydroperoxides to their corresponding alcohols and reduction in hydrogen peroxide to water	[299]
Nrf2	Detoxification of Reactive Oxygen Species	Transcription factor that binds to antioxidant response *cis*-acting response elements in the promoters of target genes	[73]

KGF: keratinocyte growth factor; ATP: adenosine triphosphatase; MMP: matrix metalloproteinase protein.

### FGFR2b signalling in tumourigenesis and tumour suppression

Keratinocyte growth factor is currently approved for use in patients with hematologic malignancies to reduce the incidence and duration of severe oral mucositis that occurs following intensive chemoradiotherapy. If KGF is to be used for a similar purpose in patients with solid tumours of epithelial origin, it must not result in enhanced growth or metastasis, or confer a cytoprotective effect on the malignant cells themselves, rendering them resistant to the therapies designed to kill them. This section reviews the current state of knowledge concerning the impact of FGFR2b signalling on tumourigenesis *versus* tumour suppression, with particular attention to the specific tissue being targeted.

Various genetic alterations occur within *FGFR2* in endometrial, ovarian, breast, lung and gastric cancer that are thought to promote tumourigenesis. Missense mutations clustered around the third immunoglobulin-like domain result in altered ligand binding specificity and activation of FGFR2 signalling [74–77]. Mutations within the tyrosine kinase domain negate an autoinhibitory molecular mechanism, resulting in ligand-independent kinase activation [77]. *FGFR2* gene amplification in breast and gastric cancer results in overexpression of FGFR2 and activation of signalling [78, 79]. C-terminal deletion of FGFR2 accompanying *FGFR2* gene amplification is because of the exclusion of the last exon from the *FGFR2* amplicon, and leads to activated FGFR2 signalling based upon constitutive phosphorylation of the FRS2 adaptor molecule [80]. Five single nucleotide polymorphisms (SNPs) located within intron 2 of the *FGFR2* gene are associated with an increased risk of breast cancer [81–84]. The mechanism associated with the increased incidence of mammary tumourigenesis in these patients remains to be elucidated, but may be associated with the alteration of various transcription factor binding sites within this region [85].

A switch from FGFR2b to FGFR2c expression occurs during the progression of bladder, prostate, salivary, and hepatocellular cancer [7, 86–88], and accompanies an epithelial-mesenchymal transition, with increased potential for invasion and metastasis [89–91]. Restoration of FGFR2b expression to malignant prostate, bladder, salivary, and hepatocellular cells results in KGF-dependent growth inhibition, reduced tumour growth, restoration of epithelial differentiation, and induction of apoptosis [86, 92–96]. Mice lacking epidermal *Fgfr2b* expression show increased sensitivity to chemical carcinogenesis, with the development of papillomas and squamous cell carcinomas bearing oncogenic *ha-ras* mutations [97]. FGFR2b mRNA expression was lower in a sample of squamous cell carcinomas (SSC) than in normal skin samples. Furthermore, KGF did not stimulate SSC cell proliferation, rather it reduced SCC cell invasion through collagen [98]. Keratinocyte growth factor also induced expression of several genes in SCC cell lines that are normally down-regulated relative to normal keratinocytes, including a number with tumour suppressor properties (*SPRY4, DUSP4, DUSP6, LRIG1, PHLDA1*). Conversely, KGF also down-regulated a set of genes that are expressed at higher levels in SSC cells compared with normal keratinocytes, including a number associated with tumour progression (*MMP13, MATN2, CXCL10, IGFBP3*) [98]. Keratinocyte growth factor is secreted from γδT cells (a subset of intraepithelial lymphocytes) within the epidermis during wound healing [99], and mice lacking γδT cells also display enhanced sensitivity to chemical carcinogens [100]. Taken together, these results indicate a tumour suppressive role for FGFR2b in the skin.

As reviewed previously, an important question regarding the potential use of KGF to minimize mucositis in patients with tumours of epithelial origin is whether KGF would have a cytoprotective effect on the tumour cells themselves, increasing their resistance to the cytoablative treatments intended to kill them [101]. The available evidence appears to suggest that in tumours in which FGFR2 is activated, such as breast, lung, and pancreas, KGF action may protect against the cytotoxic effects of radiation or chemotherapy by inhibiting the induction of apoptosis [102], [61, 71, 103–107]. On the other hand, tumours, such as squamous cell, prostate, and colorectal carcinomas in which FGFR2b activation has not been documented, appear to retain their sensitivity to cytoablative treatments in the presence of KGF [108–111]. As described above, KGF signalling through FGFR2b results in the tyrosine phosphorylation of protein subsets with well defined roles in promoting growth stimulation, as well as various novel FGFR2b-specific targets associated with the attenuation of cell growth [49]. Further analysis of phosphorylation patterns in various tumour types may provide insights about the likelihood that KGF would exhibit growth promoting or suppressive effects.

### KGF family members as presynaptic organizing molecules

A recent and hitherto unsuspected role for members of the KGF subfamily of FGFs has been their identification of their involvement in the organization of specific presynaptic terminals in the mammalian brain. Although no preclinical studies have been described in this area, the data discussed below have been included because of their novelty, and because they imply that regulation of these FGFs may have clinical relevance for the prevention of epilepsy or other neurological disorders associated with abnormal synapse formation.

Axons become specialized for neurotransmitter release at sites of precise contact with their synaptic targets, implying that target-derived factors organize presynaptic differentiation. FGF22 was identified as a major target-derived presynaptic organizing molecule from the mouse brain. FGF22 is expressed by cerebellar granule cells during the period when they receive synapses, whereas FGFR2 is expressed by pontine and vestibular neurons when their axons (mossy fibres) are making synapses on granule cells. Neutralization of FGF22 at post-natal day 3, by injecting into the lateral ventricle of the brain a recombinant fusion protein containing the FGFR2b extracellular domain linked to alkaline phosphatase, inhibited presynaptic differentiation of mossy fibres at sites of contact with granule cells [17]. Conditional post-natal inactivation of FGFR2 [112] had similar results. Keratinocyte growth factor and FGF10 are also able to promote vesicle clustering and neurite branching *in vitro*, and are expressed by neuronal subsets *in vivo* [113].

The differential formation of excitatory (glutamate-mediated) and inhibitory (GABA-mediated) synapses is critical for normal brain function. An imbalance in these synapses may result in various neurological disorders such as autism, schizophrenia, Tourette's syndrome, and epilepsy. FGF22 and KGF are expressed by CA3 pyramidal neurons in the hippocampus. The differentiation of excitatory or inhibitory nerve terminals on dendrites of CA3 pyramidal neurons is specifically impaired in mutants lacking FGF22 or KGF, respectively. Presynaptic defects are rescued by post-synaptic expression of each appropriate FGF. As might be expected from their differential roles in synaptic development, FGF22 deficient mice are resistant to epileptic seizures, whereas KGF null mice are prone to them [113]. Further analysis of KGF^−/−^ mice suggests that KGF deficiency impairs inhibitory s formation, resulting in mossy fibre sprouting and enhanced neurogenesis during development, which leads to increased vulnerability to epilepsy [114]. The differential effects of these factors might be accounted for by distinct synaptic localizations in hippocampal neurons, with FGF22 localized in glutamatergic synapses, and KGF localized at GABAergic synapses, respectively [113]. Furthermore, whereas the clustering of both glutamatergic and GABAergic vesicles was decreased in mice in which *Fgfr2* is inactivated postnatally, the decrease is more pronounced at GABAergic synapses [17, 113], suggesting that the ability of FGF22 to also signal through FGFR1b [19] might contribute to the differential effects of FGF22 and KGF. Finally, it has been reported that a SNP located in the 3′-flanking region of the *FGFR2* gene is associated with an increased incidence of schizophrenia, indicating that it may be a potential susceptibility gene for this mental disorder [115].

## Preclinical models of KGF protective and regenerative effects

Several preclinical studies have demonstrated the protective effects of palifermin in reducing chemotherapy and radiation-induced gastrointestinal injury and mortality. These striking results have been complemented by investigation of palifermin's cytoprotective and regenerative effects in a number of different tissues and organ systems after exposure to various insults. The findings of these studies are described in the following sections.

### Palliative care in the oncology setting

Preclinical data in multiple animal models of mucositis [116] provided the basis for the design and execution of clinical studies that led to the approval of palifermin to reduce the incidence and duration of severe oral mucositis in hematologic transplant settings where radiation and chemotherapy are used. Furthermore, they also contributed to the study designs for clinical trials in other contexts such as head and neck cancer (HNC) and colorectal cancer (CRC) [117–119]. Since then, the key preclinical developments relevant to the oncology setting include KGF gene therapy for oral mucositis, and the potential for new indications including cancer therapy-related xerostomia (dry mouth), diarrhoea, and bladder dysfunction.

Clinically, adverse events such as rash, erythema and pruritis of the skin and oral cavity are associated with the systemic administration of palifermin [42]. Local delivery could potentially reduce side effects and minimize the exposure of remote tumours to systemically circulating KGF. Gene transfer of KGF into salivary glands was used for local delivery of KGF into the oral cavity in a mouse model of local radiotherapy for HNC. Adenoviral vector transduction of KGF gene into salivary glands decreased the number and size of ulcers caused by radiation as assessed grossly and by morphometry. There was also a systemic benefit to the mice with the prevention of radiation-induced weight loss [120]. Xerostomia because of salivary gland hypofunction is a major complication of HNC radiotherapy [121]. In the same HNC radiotherapy model, salivary gland flow in response to pilocarpine, a common pharmaceutical treatment for xerostomia, was improved by KGF gene transfer, and this benefit was in the absence of any effect on the growth or radiation sensitivity of squamous cell carcinoma tumours in the transduced animals [120]. Because elevated levels of KGF were detectable in serum as well as the salivary glands, it is less obvious that the effects measured in this model were only a result of local KGF delivery to the oral cavity. If delivery can be optimized to reduce systemic KGF secretion, KGF gene delivery to salivary glands may alleviate pain and suffering in HNC patients by prevention of oral ulceration as well salivary gland dysfunction and xerostomia.

Systemically administered palifermin also increased the proliferation and wet weight of salivary glands in normal and irradiated mice [119, 122] and increased salivary gland flow in response to pilocarpine stimulation measured as late as 90 days after irradiation [122]. Pre, post and combined pre and post schedules of palifermin dosing relative to irradiation all showed benefit with the combination schedule being most effective. This appeared to be because of an expansion of the FGFR2b positive duct stem/progenitor cells that differentiate into acinar cells, rather than a change in radiation sensitivity of acinar cells themselves, as they did not express FGFR2b.

Diarrhoea is a complication of abdominal or pelvic radiotherapy and many chemotherapeutic agents, particularly fluoropyrimidines such as 5-fluorouracil (5-FU) and capecitabine, and irinotecan [123]. There are multiple reports on the various intestinal effects of palifermin in rats and mice exposed to chemotherapy or radiation, but diarrhoea as a symptom was measured directly in only one. In this model of irinotecan therapy, rats pretreated with single or multiple KGF dosing regimens had significantly reduced onset and severity of diarrhoea and improved mortality. Importantly, these experiments were performed in tumour-bearing rats and KGF did not adversely affect tumour growth [124]. Palifermin has been shown to reduce diarrhoea in models of IBD (see below) [125], further suggesting that this complication may be a clinically important end-point worth further evaluation in preclinical models.

Injury to the bladder also can occur with chemotherapy and abdominal or pelvic radiotherapy. Palifermin pretreatment prevented cyclophosphamide-induced hemorrhagic cystitis in rats [126] and ameliorated early functional effects of radiation-induced bladder dysfunction, as well as late effects, after single dose irradiation of the pelvic region in mice [127]. In contrast to efficacy seen previously with pre, post, and combined pre/post dosing in limiting radiation-induced oral epithelial injury [117, 118], only pretreatment dosing was effective for radiation injury to the bladder.

In summary, the wide array of agents used to treat cancer cause varying types of injury to epithelia in multiple organ systems. Many of these effects are well documented, but as novel targeted agents are approved and used in combination with older agents, new adverse event profiles and dose-limiting toxicities will emerge. Additional preclinical evaluation may be required to determine the therapeutic potential of palifermin in oncology settings where these agents are used.

### Intestinal mucosal injury

Keratinocyte growth factor is expressed in mesenchymal cells of the intestines including γδ T-cells which play an important role in intestinal growth and integrity. The γδ T-cells express KGF in activated, but not resting states [41, 128] and evidence from KGF and γδ T-cell knock out models indicates that KGF from these cells is a key factor for intestinal epithelial cell proliferation and villus growth and response to injury [41, 129]. Exogenous KGF administration causes crypt cell proliferation, mucosal thickening, increased numbers of goblet cells, and up-regulation of intestinal trefoil protein (ITF also known as Trefoil Factor Family 3 or TTF3) expression [125, 130, 131] and other effects which contribute to its protective and reparative ability in the lower gastrointestinal tract.

There are a variety of gastrointestinal maladies where loss of intestinal surface area results in malabsorption of fluid, electrolytes and nutrients, and compromise of barrier function. These conditions include IBD, necrotizing enterocolitis, intestinal ischemia/reperfusion, trauma, and bowel resection for tumours and other conditions that leave inadequate length of bowel, known as Short Bowel Syndrome (SBS). Keratinocyte growth factor homeostasis appears to be perturbed in some gastrointestinal disorders, including IBD [32, 33, 35, 132] and coeliac disease [133]. Furthermore, KGF therapy has been shown to be efficacious in animal models of IBD [41, 125, 134, 135], starvation-induced intestinal epithelial atrophy [136], intestinal ischemia/reperfusion [137] and SBS [131, 138]. Efficacy benefits were mediated by a variety of different mechanisms including crypt cell proliferation leading to crypt and villus growth [139], decreased apoptosis [140], up-regulation of goblet cells with their protective mucins and peptides [125, 131, 141, 142], and up-regulation of cytoprotective and reparative pathways [135, 142]. The above studies suggest the potential for KGF as a therapeutic for various types of intestinal insufficiency.

Most therapeutics for IBD target the immune system. However, many of the symptoms arise from erosion of the intestinal mucosa which leads to intestinal insufficiency and barrier compromise. In acute (DSS: dextran sodium sulphate) and chronic (CD4^+^CD45RB^Hi^; T-cell transfer) models of IBD, palifermin was able to ameliorate weight loss and improve survival [125]. The therapeutic effect correlated with improved gross appearance and reduced erosions of the large bowel, as well as improvement in systemic parameters. Keratinocyte growth factor gene therapy *via* oral gavage was also shown to ameliorate acetic acid-induced ulcerative colitis in rats [135]. Although these efficacy data are encouraging, further preclinical testing of palifermin in IBD models would be warranted because patients with ulcerative colitis and Crohn's disease are at increased risk for developing CRC [143] and administration of a growth factor might further elevate the risk. The IL10 knock out mouse may prove useful to address the issue of tumour stimulation by palifermin, because long-term inflammation and progression to cancer of the large bowel is common in this model [144]. Also, patients with IBD are often treated with anti-inflammatory therapeutics and the potential interaction with palifermin, which was observed to have anti-inflammatory effects in lung and GVHD (see below), should be explored.

Short Bowel Syndrome can result from massive resection of the small bowel that decreases the functional mucosal surface area [145]. Following resection, there is a complex adaptive response characterized by an increase in DNA, protein, crypt depth and villus length to compensate for loss of mucosal surface area. However, when the adaptive response is inadequate, SBS ensues with an expected survival of 15–25% in children, and 15–47% in adults [146]. Treatments for SBS include total parental nutrition (TPN), antidiarrheals, supplements, vitamins and proton pump inhibitors, all of which treat only the symptoms but not the underlying condition. Intestinal atrophy is a disorder that can be induced by abstention from eating and is observed in humans after long-term TPN [147]. Atrophy is accompanied by decreased epithelial cellularity, impaired barrier and absorptive functions and decreased antioxidant capabilities [142]. Patients on TPN or with SBS (with or without TPN) could benefit from therapy that would expand the total surface area, and/or increase the functionality of the intestinal remnant.

Palifermin has been extensively tested in models of SBS and TPN. In a rat model of SBS early adaptive intestinal growth responses such as increases in wet weight and mucosal thickness were further augmented by administration of palifermin [131]. In a mouse model of SBS where mucosal function was measured *ex vivo* in Ussing chambers, palifermin treatment following resection partially improved sodium absorption, but not other measures of barrier function such as mannitiol permeability or transepithelial resistance [138]. In models of intestinal atrophy, including fasting and TPN, studies show that palifermin is generally trophic but that there are tissue specific variations in the response to the lack of enteral nutrition and to palifermin [136, 139]. Mechanistically, palifermin prevented apoptosis and the decline in antiapoptotic Bcl-2 mRNA expression observed with TPN [140], and also improved the gut mucosal glutathione redox state in fasted rats [148]. The antioxidant benefit was in part a consequence of increased mucosal glutathione content *via* regulation of the enzymes involved in antioxidant and detoxification functions including non-selenium-dependent glutathione peroxidase and glutathione-S-transferase [142, 148]. In TPN-treated rats, significantly increased measures of plasma gastrin, PYY, total glucagon, enteroglucagon and glucagon-like-peptide-1 suggested that the palifermin effect could be mediated by these gut hormones [139].

Intestinal ischemia/reperfusion injury is encountered in many clinical settings and, like the above conditions, results in loss of intestinal epithelium and increased permeability, sometimes with drastic consequences including sepsis and multiple organ failure [149]. Experiments in a mouse model showed that KGF administered prior to ischemia reperfusion injury improved gross, morphological and histological damage, as well as measures of growth such wet weight, protein, RNA and proliferation. Intestinal epithelial apoptosis was reduced and transepithelial resistance measured *ex vivo* in Ussing chambers was improved. Tight junction protein organization (including ZO-1 and Claudin-1) was preserved by KGF, presumably contributing to maintenance of barrier resistance [137]. Studies of human tissue derived from resected bowel of patients with intestinal obstruction, in conjunction with observations in this mouse model and in human intestinal epithelial cells vitro showed that, as in the thymus (see below), IL7 likely played a role in the KGF effect, presumably through mediation of cross-talk between intestinal epithelial cells which produce IL7, and intraepithelial lymphocytes which express the IL7 receptor and endogenous KGF [149].

These preclinical data suggest that there is potential for palifermin as a therapeutic for many conditions that result in intestinal insufficiency, and a small study in humans in the oncology setting showed that patients treated with palifermin had retained intestinal barrier function and required less supportive TPN [150]. In humans, either short term or intermittent palifermin therapy post-operatively after intestinal resection, or during TPN administration, might be able to augment the body's natural adaptive response and preserve the integrity of the remnant intestine. A key question is whether the response would be sufficiently durable, as long-term effects have not been modelled in animal studies. Combination therapy with other enterotropic agents that act through different mechanisms also warrants consideration. Washizawa *et al*. [151] found differential effects of glucagon-like-peptide-2 (tedaglutide, recently approved by the FDA for SBS in adults), growth hormone and palifermin administration after intestinal resection, and recommended that combination therapy with these growth factors be evaluated. Until the issue of tumour stimulation is addressed, however, palifermin may be warranted as a chronic therapy only in settings where morbidity and mortality are very high.

### Graft-versus-host-disease and immune reconstitution

Allogeneic bone marrow transplantation (BMT) is a common procedure for the treatment of hematologic malignancies. Donor blood marrow and peripheral blood stem cells are routinely used for the reconstitution of immune function in leukaemia and lymphoma patients after radiation and/or chemotherapy. Graft-versus-host-disease is a common complication of allogeneic BMT in which functional immune cells in the donor marrow recognize histocompatibility antigens of the host tissue and mount an immunologic attack. Activated donor T-cells produce an excess of cytopathic molecules, including TNF-α and IFN-γ, which results in tissue damage to the skin, GI tract, liver, lung and immune system.

Graft-versus-host-disease can be effectively prevented by depletion of T-cells from the donor, but this results in the loss of graft-versus-leukaemia (GVL) effect, greater risk of engraftment failure, and cancer relapse [152]. Another common prophylaxis is immunosuppression of the host, but this puts the patients at greater risk of infection. Damage to the GI tract is thought to play a pivotal role in the pathophysiology of GVHD by augmenting the release of cytokines that promote inflammation and cytotoxic immune cell activities [153, 154]. Therefore, an alternative approach to the prevention of acute GVHD is to retain the mature T-cells within the graft, but disrupt the amplification of inflammatory cytokine effectors. Thus, it was originally proposed that KGF might be useful in this regard by reducing injury to the GI tract, particularly damage resulting from the chemoradiotherapy conditioning regimens that precede BMT, thereby diminishing the ensuing inflammatory cascade [153, 155, 156].

Several BMT models have demonstrated a beneficial role for palifermin in limiting GVHD when administered either pre- [157–160], or post-transplant [161]. Palifermin enhanced the survival of transplant recipients, whereas at the same time ameliorating GVHD-related pathologic changes in liver, lung, skin, and GI tract [157, 159]. However, subsequent investigations of BMT in unconditioned mice have shown that palifermin reduced GVHD, even in the absence of a mucotoxic conditioning regimen [162, 163]. Palifermin treatment reduced the *in vivo* allo-response, and altered the plasma cytokine levels during acute GVHD. These alterations were manifested by the development of a mixed Th1/Th2 cytokine ratio, in which Th2 cytokines such as Il-4 and Il-13 predominated [162–164]. Thus, these studies imply an immunomodulatory mechanism of palifermin action that is in addition to, and independent of, its beneficial direct cytoprotective effects on epithelial tissues against damage caused by radiation, cytotoxic therapy, and/or GVHD.

The outcome of BMT is ultimately dependent upon a successful and comprehensive reconstitution of the immune system with a broad T-cell receptor (TCR) repertoire, which requires the *de novo* generation of T-cells in the thymus. The thymus is a specialized organ of the immune system, whose primary function is the production and maturation of T lymphocytes (T-cells) from hematopoietic precursors (thymocytes) derived from the bone marrow. Mature T-cells emigrate from the thymus and constitute the peripheral T-cell repertoire, responsible for directing several aspects of the adaptive immune response. The stock of T lymphocytes is built up early in life, and the function of the thymus diminishes in adults. Involution of the thymus in the elderly is associated with the loss of immune function, susceptibility to infection, and the development of malignancies. The thymic stromal compartment is composed of a complex three-dimensional network of epithelial cells with distinct phenotypes [165]. Thymic epithelial cells (TEC) mediate discrete functions including the elaboration of cytokines to promote the attraction of hematopoietic precursors to the thymus, mediation of positive T-cell selection through binding to surface TEC major histocompatability complex (MHC) molecules, and participation of medullary TECs in negative T-cell selection by displaying self antigens to developing T-cells thus instructing self reactive T-cells to undergo apoptosis. Cytoablative conditioning regimens used to treat malignancies also destroy thymic architecture, resulting in diminished thymic output and function that results in impaired immunity following BMT.

Keratinocyte growth factor is expressed within the thymus, both by mesenchymal cells and by T-cells, at specific developmental stages, whereas FGFR2b is expressed by TECs. Embryonic FGFR2b^−/−^ mice have profound defects in thymopoiesis, with diminished thymic cellularity resulting from the impaired proliferation and differentiation of TECs [166]. The thymus of FGF10^−/−^ mice is similar to that of FGFR2b^−/−^ mice, although their thymopoietic effect is less severe [166]. In contrast, KGF^−/−^ mice exhibit normal thymic organogenesis and post-natal thymopoiesis [161]. During thymic organogenesis, KGF stimulates TEC proliferation before hematopoietic cells seed the thymic primordium [167, 168]. Keratinocyte growth factor induces the expansion of immature TECs, and promotes their differentiation. The resulting stromal changes initiate an expansion of immature thymocytes, and permits regular T-cell development [169]. Keratinocyte growth factor activates signalling in TECs *via* the p53 and NF-κB pathways, resulting in increased transcription of several genes required for TEC function and T-cell development, including bone morphogenic proteins 2 and 4, Wnt5b and Wnt10b [170]. In addition to thymic mesenchymal cells, mature αβ^+^ thymocytes also produce KGF, which contributes to the expansion of thymic medullary epithelial cells [167]. In contrast, KGF expression was not detectable in thymocyte precursors, peripheral αβ^−^ T-cells [167]. Palifermin is able to compensate for age-related thymopoietic insufficiency in elderly mice, resulting in increased numbers of naïve CD4 T-cells in the periphery, and improved T-cell-dependent antibody production [171]. Palifermin treatment increased TEC proliferation, resulting in the reorganization of cortical and medullary architecture [171]. Finally, whereas KGF^−/−^ mice exhibit normal thymic development, they display defective thymic recovery following sublethal radiation, indicating that KGF plays a significant role in post-natal thymic regeneration following injury [161]. Such a role for KGF is consistent with other experimental findings, which suggest that KGF has a specific role in cytoprotection and repair, not redundant with FGF10 or other FGFs [41].

Palifermin enhances thymopoiesis following experimental BMT by cytoprotection of specific TEC populations within the thymic cortex and medulla, as well as preserving TEC function [158, 169, 171]. IL7 is an essential cytokine for T-cell development and survival, and is a key regulator of T-cell homeostasis in the periphery [99, 172], The thymopoietic effects of palifermin required TEC-thymocyte cross-talk mediated by IL7 signalling [158]. In a CD8^+^ T-cell dependent mouse model of GVHD directed against minor histocompatibility antigens, it was found that pathogenic donor CD4^+^ T-cells developed from engrafted hematopoietic stem cells in the stressed and impaired thymus during acute GVHD and mediate the transition to chronic GVHD [173]. Palifermin treatment improves the restoration of thymic dendritic cells, which are involved in the deletion of self-reactive T-cells, and prevented the *de novo* generation of pathogenic CD4^+^ T-cells [173]. These results suggest a mechanism whereby palifermin prevention of thymic damage may lead to a direct amelioration of chronic GVHD. Palifermin enhancement of T-cell reconstitution has also been demonstrated in rhesus macaques following autologous CD34^+^ peripheral blood progenitor transplantation and conditioning with myeloablative total body irradiation, thus providing a primate model for clinically relevant specific immune responses [174].

Regulatory T-cells are a specialized subpopulation of T-cells that act to suppress activation of the immune system and thereby maintain immune system homeostasis and tolerance to self-antigens. Regulatory T-cells come in many forms, including CD4^+^CD25^+^Foxp3^+^ regulatory T-cells (Treg), which are thought to play an important role in the prevention of GVHD [175–179]. Palifermin administration was shown to increase peripheral CD4^+^Foxp3^+^ Treg numbers *via* two independent mechanisms [180]. The first wave of expansion, occurring within 4 days of palifermin administration, was the result of selective peripheral expansion, whereas a second wave from day 10 onwards is a result of enhanced thymic output of newly developed Treg [180]. The effects of palifermin on Tregs have been demonstrated to account in part for the immunomodulatory effects of palifermin after BMT. Treatment with palifermin of congenic wild-type mice that served as T-cell provider for T and B cell-deficient RAG-1^−/−^ mice, significantly improved engraftment and reduced graft rejection in graft recipients [181]. When Scurfy mice, which lack Foxp3^+^ Treg, were used as T-cell providers, the ability of palifermin to improve engraftment was lost [181]. Thus, palifermin appears to improve allogeneic bone marrow engraftment through a CD4^+^Foxp3^+^ Treg-dependent mechanism.

As described above, palifermin facilitates alloengraftment and abrogates GVHD-induced lethality in murine BMT recipients. However, as it fails to completely protect medullary TECs and to restore CD8^+^ T-cell numbers and function following BMT, various studies have been performed to assess the efficacy of combining palifermin treatment with other promising therapies for GVHD. The tumour suppressor gene, p53, is activated to induce apoptosis and/or growth arrest following genotoxic stress, resulting in the elimination of damaged cells from the organism [182]. A small molecule (pifithrin-β; PFT-β), which was isolated on the basis of its ability to reversibly block p53 function *in vitro* and *in vivo* [183], abrogates apoptosis in epithelial tissues and protected mice from lethality following excessive doses of total body irradiation [183]. Combined administration of palifermin and PFT-β before BMT additively restored numbers of cortical and medullary TECs, and improved thymic function following BMT, resulting in higher numbers of donor-derived, naïve peripheral CD4^+^ and CD8^+^ T-cells [184]. Androgen receptors (ARs) are expressed on TECs, certain thymocyte subsets, and mature T-cells [185–189], and the thymic atrophy that occurs with advancing age has been partly attributed to physiological changes in sex steroid hormone production. Physical castration of aged mice results in restoration of thymic size and function to prepubertal levels, and mice castrated pre-BMT exhibit enhanced thymopoiesis and peripheral T-cell numbers relative to control recipients [190–195]. Pre-BMT androgen blockade *via* chemical castration, when used in conjunction with palifermin treatment, led to the restoration of thymic architecture, number, and subset distribution of TECs [195]. Furthermore, there was an additive effect upon the restoration of thymopoiesis, thymic output, and recovery of peripheral naïve T-cell numbers [195]. Thus, the use of either transient p53 inhibition or androgen blockade, in conjunction with palifermin treatment may represent novel approaches to accelerate thymic T-cell recovery and function following BMT.

Despite the encouraging results regarding the effect of KGF in reducing GVHD and promoting immune reconstitution in preclinical models, thus far several clinical trials of palifermin conducted in patients undergoing allogeneic stem cell transplants have failed to demonstrate significant advantage in the reduction in GVHD, control of infection or long-term survival (see accompanying review article in this series). Another possible application of palifermin in this general clinical area might be in the setting of hematopoietic stem cell transplants using umbilical cord blood. The use of cord blood stem cells in allogeneic stem cell transplants has increased recently because of the low incidence of GVHD and the fact that they are less immunogenic so that the requirement for HLA matching is less stringent [196]. However, the success of these transplants is currently not as good as autologous or conventional allogeneic transplants because the immune cells are very immature, leading to delayed immune reconstitution, and there is a greater risk for recurrence of the underlying malignancy. In this regard, palifermin was used to investigate thymopoiesis in a mouse allogeneic full-term foetal blood cell transplant model with platelet concentrate support to minimize bleeding problems. Palifermin administration resulted in increased donor-derived T and natural killer T-cells in the spleens of mice undergoing UCB stem cell transplant, and also improved thymic function [197]. Furthermore, pretreatment with palifermin prior to cord blood stem cell transplant helped prevent relapse of leukaemia, indicating enhanced GVL function [197].

### Pulmonary disease

Keratinocyte growth factor/FGFR2b expression patterns in lung reflect those seen in other epithelial tissues with receptor expression restricted to the tracheal and pulmonary epithelium and the ligand expressed in pulmonary mesenchyme [198]. Although lungs in KGF^−/−^ mice appeared normal [22], KGF had profound effects on the developing lung when it was constitutively over-expressed *via* a surfactant protein C promoter. Branching morphogenesis was disrupted and the thoracic cavity filled with dilated cystic structures that resulted in embryonic lethality [199]. However, controlled administration of palifermin to neonatal and adult animals had a more benign effect, whether given intratracheally (IT) or intravenously (IV): a dose-dependent stimulation of alveolar and bronchial epithelial proliferation [200, 201]. Studies show that KGF also regulates lung barrier function including ion and fluid transport in normal and injured tissue [202–209] as well as surfactant homeostasis [37, 210–212]. Besides its direct effects on the alveolar epithelium, it may also influence integrity of the pulmonary endothelial barrier *via* regulation of paracrine factors such as VEGF [213]. Keratinocyte growth factor and/or FGFR2b are up-regulated in lung injury models [25, 27, 214] and in human volunteers with lung damage, suggesting that it has a role in repair processes [215]. Palifermin has been tested in numerous animal models of lung damage or dysfunction, as well as in human lung injury settings, as summarized in the following sections.

#### Acute lung injury (ALI)

Acute lung injury is a clinical syndrome associated with a variety of conditions including sepsis, pneumonia, aspirations and trauma. It is characterized by injury to the alveolar epithelial barrier, impaired surfactant homeostasis and decreased alveolar epithelial fluid transport [216] resulting in the development of non-cardiogenic pulmonary edema and need for mechanical ventilation. Current treatment consists of protective lung ventilation with a conservative fluid strategy but mortality remains high and new therapeutic strategies are needed [217]. Numerous experimental studies suggest that palifermin is one of the emerging candidates for therapeutic intervention with potential to repair the damaged lung [218].

Keratinocyte growth factor has been evaluated in multiple different models of acute lung injury. In adult rats, where palifermin was administered IT prior to exposure to hyperoxia to induce lung injury, there was a dose-dependent improvement in mortality that correlated with improved lung injury histology [201]. Proliferation of alveolar type 2 (AT2) cells was observed in the KGF-treated hyperoxic rats, but the hypothesis that other mechanisms of protection might include antioxidant enzyme activity were not borne out as no changes were measurable in superoxide dismutase (SOD), MnSOD and Cu/ZnSOD. Palifermin given IV to adult mice prior to or during hyperoxic exposure attenuated measures of damage including increases in lung wet weight, and preserved both the normal epithelial and endothelial histology [219]. AT2 proliferation was not observed in the mice, but the induction of apoptosis-related genes including p53, Bax and Bcl-x was reduced and fibrinolytic activity was restored, possibly *via* suppression of plasminogen activator. Improved survival was observed with IT KGF gene therapy in mice, and correlated with other histological and physiological benefits such as improved arterial oxygen tension [220]. In neonates, exposure to hyperoxia is a model of bronchopulmonary dysplasia (BPD) in premature infants. Systemic palifermin treatment increased survival of newborn rat pups when given during exposure to hyperoxia [37]. This survival benefit was associated with reduced neutrophil influx into bronchoalveolar lavage (BAL) fluid and protection against DNA loss, but not with cell proliferation, apoptosis or production of lung surfactant. *In vitro* experiments in the same report suggest that palifermin was providing alveolar protection, rather than enhancing repair, by the prevention of non-apoptotic cell death. Similarly, palifermin improved survival in premature rat pups exposed to hyperoxia. In this model pulmonary hypertension was prevented but pulmonary fibrosis (PF) was not [221].

Other agents also have been used to model ALI. In alpha-naphthylthiourea (ANTU) induced-injury, palifermin attenuated ANTU-induced oedema formation by potentiating alveolar fluid clearance. This effect was mediated through AT2 hyperplasia and increased sodium–potassium-adenosine triphosphatase (NaK-ATPase) activity [202]. In an acid-installation injury model in rats, palifermin pre-treatment reduced lung capacity impairment. It decreased morphological damage, and reduced inflammatory cells in BAL fluid, as well as procollagen mRNA and hydroxyproline accumulation, indicating the potential to prevent subsequent PF [222]. Anti-inflammatory effects of palifermin were also seen in mice exposed to acid as KGF reduced MIP-2α concentration and neutrophil influx [223]. Systemic administration of oleic acid induces ALI in many species. Treatment of mice with IT palifermin 2 days prior to oleic acid exposure resulted in significant improvements in arterial blood gases and lung compliance [224]. However, these benefits did not correlate with other measures of tissue damage (*e.g*. protein exudation, cellular infiltrates), possibly because of the relatively short time course of the study (1 hr). In models of pneumonia, palifermin restored normal alveolar epithelial fluid transport during the acute phase and lung liquid clearance in early and late-phases, while improving host responses, in part through GM-CSF-stimulated macrophage activation [206, 225].

A few studies have been performed to evaluate the role of KGF in humans with ALI. Endogenous KGF has been detected in pulmonary oedema and BAL fluid from patients with ALI, but its clinical significance is uncertain [216, 226]. Bronchoalveolar lavage and alveolar fluid either inhibit or have little effect on the expression of KGF by human fibroblasts [227, 228]. In a human *ex vivo* lung perfusion model of lipopolysaccharide (LPS)-induced injury, KGF present in mesenchymal stem cell conditioned medium improved the rate of alveolar fluid clearance and consequently reduced pulmonary oedema. When the stem cells were pre-treated with KGF siRNA, 80% of the protective effect was abolished, and instillation of KGF alone into the lung lobe partially restored fluid clearance, suggesting that other soluble factors probably contribute to fluid clearance [217]. Interestingly, the beneficial effects were seen when the KGF-containing medium was given 1 hr after the lung had been exposed to LPS. Palifermin has been tested in healthy human volunteers who inhaled low dose LPS, which is known to induce pathophysiological changes, characteristic of ALI. Administration of palifermin at 60 μg/kg/day IV for 3 days prior to the LPS exposure led to increases in the concentration of mediators associated with epithelial repair such as MMP-9 and IL-6 [229]. These results prompted a prospective, randomized, placebo-controlled, double-blind phase 2 clinical trial to determine whether palifermin given within 48 hrs of onset of ALI would improve physiological markers and clinical outcome (ISRCTN95690673). Sixty patients will be randomized to 60 μg/kg palifermin or placebo daily as a bolus IV injection for up to 6 days. Surrogate clinical outcome of pulmonary physiological function (including oxygenation index and respiratory compliance) and systemic organ function as measured by SOFA score [230] will be assessed. Indicators of pulmonary and systemic inflammation, alveolar epithelial and endothelial function, protease:antiprotease balance and lung extracellular matrix degradation and turnover also will be studied.

Although the beneficial effects of palifermin in animal and human ALI models typically have involved its use prior to the toxic exposure, one case report suggested it might have value when given after the onset of tissue damage [231]. A patient with ALI and damage to the upper aerodigestive system survived ingestion of what was expected to be a lethal amount of paraquat when it was followed by the treatment with palifermin and clarithromycin.

#### Pulmonary fibrosis

Pulmonary fibrosis is characterized by alveolar epithelial cell injury, interstitial inflammation, fibroblast proliferation and collagen accumulation within the lung parenchyma [232]. It can occur in a variety of clinical conditions and in response to a wide range of agents including chemotherapy and radiotherapy. Keratinocyte growth factor therapy has been shown to be efficacious in several bleomycin-induced models of PF whether delivered as palifermin IT [233–235], as IT gene therapy [236], or *via* bone marrow stem cells expressing inducible KGF that home to lung tissue after IV injection [232]. Benefits of therapy included maintenance of body weight [235], decreased mortality [233, 236] and improved lung function [236]. *In situ*, palifermin reduced edema [237], neutrophil and protein infiltrates in BAL fluid [238] and mitigated fibrosis [233, 236, 238]. The effects appeared to be mediated by multiple mechanisms including proliferation of Clara and AT2 cells [237, 238], increased surfactant protein gene expression [235, 236, 238], and decreases in the fibrogenic cytokines TGF-β and PDGF-BB [237] as well as in the attenuation of collagen types I, II [235] and hydroxyproline [233] content.

The development of PF in response to radiation has a longer time course compared with bleomycin instillation (12 weeks *versus* 3 weeks [233]). A single IV dose of palifermin delivered after fractionated radiotherapy resulted in functional improvement. Severity of lung fibrosis was reduced, probably because of down-regulation of the TGF-β pathway [239]. In experiments using single dose radiation, KGF reduced fibrosis when given as palifermin IT prior to radiation [233], and *via* IT gene therapy when improvement appeared to be mediated by antioxidant activity, as well as up-regulation of surfactant proteins and down-regulation of TGF-beta [240].

#### Other pulmonary indications

Keratinocyte growth factor therapy has been evaluated in lung transplant models with mixed success. Palifermin treatment of isogenic donor rats prevented intra-alveolar oedema, which was attributable to increased production of surfactant protein C and corresponding improvement in surfactant function [241]. These measures were made 24 hrs after transplant. In contrast, palifermin did not improve lung allograft survival when assessed at a 4-day time-point in either of two allogeneic models, perhaps because of an enhanced allogeneic stimulus because of a combination of AT2 hyperplasia and increased expression of MHC class II antigens [242]. Palifermin treatment improved graft status in a syngeneic model of tracheal transplantation as assessed histologically, at least in part through an antiapoptotic effect on the donor cells. In addition to its direct effects on the airway epithelia, palifermin also increased by almost threefold the homing of circulating epithelial progenitor cells (expressing FGFR2b, cytokeratin 5 and CD45) from the bone marrow to the healing airway epithelium [38]. In another surgical setting, pneumonectomy, endogenous KGF and FGFR2b are up-regulated [214], and palifermin delivered IP [243] as well as KGF gene transfection [214] each augmented after pneumonectomy lung growth *via* proliferative effects.

The utility of KGF has also been examined in models of other pulmonary diseases. Emphysema is a chronic obstructive pulmonary disease, largely attributed to cigarette smoking, featuring an abnormal dilation of distal airspaces combined with destruction of alveolar walls [244]. In a model of emphysema induced by the instillation of elastase into mice, palifermin reduced pulmonary inflammation, activation of MMPs and alveolar cell DNA damage. Palifermin had multiple anti-inflammatory effects including a decrease in the levels of inflammatory cytokines (CCL2, CXCL2) and adhesion molecules (ICAM-1, VCAM-1) [245]. In a rat model of chronic asthma induced by the administration of ovalbumin, palifermin was given IV after epithelial lesions were already present, but before the final ovalbumin challenge, and was found to reduce leakage along with neutrophil and leucocyte infiltration. Epithelial integrity was improved and correlated qualitatively with expression of the junctional proteins ZO-1 and β-catenin in epithelial cells and vascular walls [208]. Exposure of the airways to environmental toxins can be modelled by systemic injection of naphthalene. Selective injury of the Clara cells occurs as a consequence of its conversion into a cytotoxic intermediate by the xenobiotic enzyme CYP2F2. Palifermin delivered by oropharyngeal aspiration prior to naphthalene exposure prevented impairment of lung function associated with the loss of Clara cells. Palifermin pre-treatment enhanced proliferation, protected cell membrane integrity and specifically down-regulated CYP2F2 as demonstrated *in vivo* and *in vitro* [246].

Taken together the studies discussed above comprise a large body of evidence that palifermin may have therapeutic potential for a variety of human pulmonary disorders. Most animal studies indicated that palifermin's benefits are dependent on pre-injury dosing that presumably creates a functional tissue reserve to facilitate protection, although some studies revealed shorter term cellular responses to palifermin that also may have clinical benefit. Typically the onset of human lung injury cannot be anticipated, except in the setting of cancer therapy or lung transplantation. This might limit the practical application of palifermin. However, Ware and Matthay [215] point out that, in contrast to the pre-clinical studies where KGF is tested only as a single agent, other supportive clinical care may provide a prolonged interval for palifermin to exert its therapeutic effects.

### Dermatologic applications

#### Wound healing

The observations that KGF was dramatically up-regulated following cutaneous injury in mouse and human full-thickness wounds [30, 31] as well as after tissue damage in the models of surgical bladder injury [26], chemically induced kidney injury [29], exposure of neonatal lungs to hyperoxia [27], and acute lung injury resulting from bleomycin injection [25], suggested that KGF participated in a wide variety of epithelial preservation and/or repair processes. Further evidence for such a role was provided by reports that KGF was up-regulated in some human inflammatory diseases, including psoriasis [34, 247] and IBD [32, 33, 35]. Finally, several KGF responsive genes are up-regulated at sites of injury and have presumed roles in wound repair (Table 1).

Based upon such observations, experiments were performed with KGF in animals to determine whether its topical application to the skin could stimulate epidermal wound repair. Keratinocyte growth factor increased the rate of re-epithelialization and epidermal thickness in partial-thickness wounds of the porcine epidermis [248] and wounds extending through the cartilage of rabbit ear [249]. Keratinocyte growth factor also increased epidermal thickness and the rate of follicular proliferation in porcine models of full- and partial-thickness burns. However, the magnitude of these effects was not considered sufficient to warrant clinical development [250]. In general, the clinical success of growth factors administered as recombinant proteins to enhance wound repair has been disappointing, despite their apparent efficacy in animal studies [251, 252]. It has been postulated that the bioactivity of the recombinant proteins is rapidly diminished because of elevated concentrations of MMPs and other myeloid cell-derived proteinases present in the wound environment [253, 254]. Furthermore, bolus administration does not keep the protein localized to the wound area and necessitates large amounts of growth factor that may have harmful side effects such as vascularization of non-target tissues or stimulation of tumour growth [255]. Thus, several alternative methods are currently being developed in efforts to enhance the tissue specificity of recombinant proteins, including the use of biomaterials to achieve controlled and localized release, as well as various gene transfer systems. In the following section, we shall review current literature focusing upon the use of such methods to enhance KGF efficacy in promoting wound healing.

A biomimetic approach has been used to investigate cell-controlled delivery of KGF. For this purpose, a matrix-binding peptide that contains two domains, one recognized by the transglutaminase factor XIII and the other containing the fibrin binding site of α2-plasmin inhibitor, was covalently attached to free amines on the surface of the KGF molecule. Upon activation by thrombin, factor XIII acts on fibrin to form γ-glutamyl-ε-lysyl amide cross-links between fibrin molecules to form an insoluble clot. Thus, during coagulation, the transglutaminase activity of factor XIII recognizes one domain of the hybrid molecule, resulting in its incorporation into the fibrin matrix at high concentration. Subsequent, cell-mediated activation of plasminogen to plasmin degrades the fibrin matrix and cleaves the hybrid peptide at the fibrin binding site to release a modified, yet still biologically active, KGF molecule into the local microenvironment [256]. Treatment of cutaneous wounds in human bioengineered skin engrafted onto athymic mice with fibrin gels containing this KGF derivative resulted in enhanced wound closure. In contrast to topically applied KGF, fibrin-bound KGF persisted in the wound environment for several days and was released gradually [256]. Another study examined the wound healing efficacy of a gelatin-based, interpenetrating polymer network (IPN) that contained poly(ethylene glycol) (PEG)-ylated arg-gly-asp and soluble KGF. While there did not appear to be a significant difference in the rate of wound contraction between the KGF containing IPN and unmodified IPN controls, the KGF IPN acted as a tissue scaffold preventing the entry of foreign bodies, and resulted in increased cellularity and extracellular matrix organization compared to wounds treated with IPN only [257]. *Aloe vera* is a succulent xerophtye in which the innermost part of the leaf consists of large, thin-walled parenchyma cells that retain water in the form of viscous mucilage for storage purposes, and which has long been used for its therapeutic properties. The use of cell wall fragments derived from this gel to bind KGF in microsheets has been described for wound healing applications, although the efficacy of this technique has not been reported [258].

The FGF binding protein (FGF-BP), originally shown to bind and activate FGF1 and FGF2, also interacts with KGF, FGF10 and FGF22, and enhances the activity of low growth factor concentrations. Furthermore, expression of FGF-BP is increased following injury to murine skin, particularly in keratinocytes [259]. Thus, up-regulation of FGF-BP following cutaneous injury seems likely to promote epithelial repair by stabilizing KGF and possibly providing protection from proteases in the wound environment. Use of this molecule, perhaps its incorporation into biomaterials, might augment the activity of KGF in wound healing applications.

Several methods have been developed for gene transfer. In rats with a full thickness scald burn, subcutaneous injection of a KGF cDNA liposomal complex resulted in significantly improved epidermal regeneration, because of increased keratinocyte proliferation and decreased apoptosis [260]. Liposomal KGF gene transfer was associated with increased levels of insulin-like growth factor I, insulin-like growth factor binding protein 3, and collagen IV, and decreased levels of TGF-beta in wound tissue [261]. The use of electroporation to enhance the cellular uptake of microinjected naked KGF cDNA has also been investigated as a tool to enhance wound healing. It has been postulated that the application of an electric field of sufficient strength across a tissue causes transient pores to open in the lipid bilayer of cell membranes, thereby increasing permeability to the cDNA molecules [262]. Electroporation with KGF cDNA enhanced wound healing in a diabetic mouse model [263] that featured markedly slower wound healing than normal and delayed KGF expression following injury [264]. Transfection of the KGF cDNA by electroporation also had a beneficial effect in a rat sepsis model of wound healing [265]. Another study assessed various methods of virus-mediated KGF delivery to promote cutaneous wound healing in human bioengineered skin engrafted onto immunodeficient mice [266]. These included KGF gene transfer by intradermal adenoviral injection, adenoviral vector immobilized in a fibrin carrier, and adenoviral transduction of human fibroblasts embedded in a fibrin matrix. The first two strategies exhibited substantial variability within each group with regard to KGF expression, but when achieved, enhancement of re-epithelialization was observed. The use of KGF overexpressing fibroblasts provided the most reproducible expression of KGF, which was associated with a significant improvement in the overall healing process [266]. The authors suggested that this might prove to be the most promising method for clinical use since it avoids the uncertainties of *in vivo* cell targeting as well as direct patient exposure to viral vectors, and offers the possibility of delivering biologically active proteins in a sustained manner to improve the chance of therapeutic success. A similar approach involved the use of HaCaT keratinocytes stably expressing high levels of KGF to enhance the healing of superficial second degree burns generated on pig skin, and indicated that engraftment of KGF expressing HaCaT cells significantly reduced the time required for complete re-epithelialization compared to control cells [267].

#### Epidermolysis bullosa

Epidermolysis bullosa (EB) is one of several types of skin-blistering disorders with considerable morbidity and mortality. Although EB is a clinically and genetically heterogeneous syndrome, it is now categorized into four main subtypes according to the level of skin cleavage: EB simplex, junctional EB, dystrophic EB and Kindler Syndrome [268]. Specific mutations in at least 17 genes have been identified that are thought to contribute to the pathogenesis of the EB subtypes. Epidermolysis bullosa simplex is associated with various mutations in the genes encoding keratin (K) 5 and K14 [269], whereas in junctional EB the majority of mutations have been found in the *LAMB3* gene (coding for the beta-3 subunit of laminin), resulting in small and sparse hemidesmosomes [270]. The generalized recessive dystrophic EB is one of the most severe forms and occurs as a result of mutations in COL7A1 (collagen type VII, chain A1), affecting both the dermal fibroblasts and basal keratinocytes. It is clinically manifested as extensive mucocutaneous blistering, esophageal strictures, mutilating scars, life-threatening infections and aggressive squamous cell carcinoma [271]. A variety of therapies that have been tested include gene therapy, protein replacement therapy and fibroblast and fibroblast and stem cell therapies [272], with early data from stem cell transplant showing promise.

The epithelial growth and reparative effects of KGF seem to be suited for therapy in the EB settings where the overall stimulation of epidermal keratinocyte proliferation and subsequent thickening of the epithelial layer might provide some protection from injury and/or aid in repair of skin and mucosal lesions. In a porcine model of epithelial wound healing, KGF increased the number of serrated basal cells associated with increased deposition of collagen fibres in the superficial dermis. Electron microscopy documented better developed hemidesmosomes and thicker bundles of tonofilaments in the serrated cells [248]. In mice, palifermin treatment increased the size and number of keratin granules and desmosomes in the esophageal epithelium [117]. Whether such biological effects would translate into epidermal tissue benefits could be tested in preclinical models of EB, particularly models of simplex or junctional EB settings where dysregulation of keratin and desmosomal component production occurs. For example, Lamc2^jeb^ mutant mice have reduced levels of LAMC2 protein and develop a progressive blistering disease that closely resembles a sub-type of junctional EB (generalized non-Herlitz) [273].

The rational for testing of palifermin in models of dystrophic EB is more complicated. The dermal fibroblasts would not respond to palifermin therapy, at least directly, as they do not express the KGF receptor. Early clinical data have shown that stem cell transplantation is a promising therapy for EB but the risk/benefit ratio of the conditioning regimen, which relies on immunomyeloablative chemotherapy, was such that it was only used in severe disease of recessive dystrophic EB [271]. Although the conditioning regimens now being used for EB are less intensive, palifermin might prove useful as an adjunct to the stem cell transplant procedure by ameliorating mucositis. In addition, it also has the potential to directly impact the underlying disease in the epithelium.

One of the most common complications of EB is the occurrence of skin cancer [274]. Although in some types of cancer, KGF signalling is thought to be associated with tumourigenesis, the opposite appears to be the case in skin cancer where studies indicate a tumour suppressive role for FGFR2b in the skin (see above). Therefore, therapy with KGF in the patients with EB setting would seem unlikely to increase the risk of developing cancer. In conclusion, given the well documented effects of KGF on keratinocyte biology, careful evaluation of palifermin in available pre-clinical models could be of benefit in defining a potential course of therapy for humans afflicted with this painful and life-threatening disease.

### Urothelium

The urothelium comprises the epithelial lining of much of the urinary tract, including the kidneys and bladder. In KGF^−/−^ mice, the developing ureteric bud and mature renal collecting system were markedly smaller than in kidneys from wild-type mice, and mature kidneys in KGF^−/−^ mice had 30% fewer nephrons than wild-type kidneys [23]. *In vitro* experiments demonstrated that KGF augmented ureteric bud growth and increased the number of nephrons that formed in rodent metanephric kidney organ cultures [23]. Conditional targeting of FGFR2 expression in developing ureteric bud tissue resulted in aberrant ureteric bud branching, as well as stromal mesenchymal patterning defects [275]. The bladder urothelium in KGF^−/−^ mice was markedly thinner than that of wild-type mice, and lacked the intermediate layers present in normal animals [24]. Primary urothelial cell cultures maintained without KGF stopped dividing and expressed markers associated with terminally differentiated umbrella cells [24]. Systemic administration of palifermin resulted in the rapid proliferation of transitional epithelium in the rat bladder [276]. Rhesus monkeys treated with palifermin for 7 days also showed a strong proliferative response in the urothelium of the bladder and renal pelvis, as well as the collecting ducts of the kidney [276].

Keratinocyte growth factor mRNA expression increased several fold following surgical bladder injury in rats [26], indicating a possible role as a mediator of bladder repair following various insults. Keratinocyte growth factor transcripts were also increased throughout the kidney following the induction of proximal tubule damage by *S*-(1,1,2,2-tetrafluoroethyl)-L-cysteine (TFEC). The FGFR2b transcript level was high in the papilla and medulla of the undamaged kidney, but increased further following TFEC administration [29]. These results suggest the activation of a paracrine loop whereby increased mesenchymal expression of KGF regulates FGFR2b-expressing proximal tubule epithelial cell growth and repair following chemical damage. In a mouse model of ischemia-reperfusion injury (IRI), it was demonstrated that FGFR2b containing urothelial cells located in the deep cortex at the corticomedullary junction were the first to proliferate following injury. Palifermin induced proliferation of these urothelial cells following *in vivo* administration to control mice, whereas inhibition of FGFR2 by an antisense oligonucleotide inhibited urothelial proliferation following IRI. Furthermore, IRI resulted in FGFR2 phosphorylation, as well as up-regulation of KGF [277]. The FGFR2b mediated proliferation of urothelial cells precedes the proliferation of FGFR2b-negative tubular cells, raising the important question as to whether this represents an important first step in the repair of tubular cell lesions following IRI [277].

Palifermin pre-treatment almost completely ameliorated cyclosphosphamide (CP)-induced ulcerative hemorrhagic cystitis in rats, with the protective effect thought to be because of the rapid proliferation of urothelial cells during and after chemotoxic injury, as well as cytoprotective mechanisms [126]. Administration of KGF prior to CP treatment maximized its protective effects. Hemorrhagic cystitis occurs sporadically in patients after radiation therapy, but is most commonly observed after CP treatment or its more urotoxic derivative, ifosfamide.

### Pericardium

The pericardium is a double-walled sac that contains the heart and the roots of the great vessels. Pericardial adhesions, which represent an attachment of the pericardium to the heart muscle, can form after surgical procedures, trauma or inflammation and may restrict the action of the heart. Injury to mesothelial cells, which comprise the membrane that lines the pericardium, impairs the fibrinolytic capabilities of the mesothelial membrane and promotes adhesion formation [278]. Keratinocyte growth factor has been shown to stimulate the proliferation of mesothelial cells [279, 280]. Therefore, it was postulated that use of palifermin could hasten pericardium recovery following surgery or trauma, leading to improved fibrinolytic function and reduced adhesion formation. In a porcine model, palifermin administration following surgical induction of adhesions significantly reduced the intensity of post-operative adhesions, and facilitated re-operation [281]. In a subsequent study, palifermin was used in conjunction with carboxymethyl chitosan, a biopolymer with properties similar to extracellular matrix, and which previously had been shown to decrease adhesion formation by providing a barrier to separate the visceral and parietal membranes. This combination therapy brought about a synergistic decrease in post-operative adhesion formation [282]. Thus, the use of palifermin to enhance mesothelial repair and promote its fibrinolytic potential, might represent a promising antiadhesion therapy worthy of further investigation.

## Conclusions and perspectives

Keratinocyte growth factor possesses many attributes required of a molecule that plays an important, if not crucial, role in mediating epithelial tissue repair following injury. It is up-regulated following tissue injury, and it acts to strengthen the integrity of the epithelial barrier *via* multiple mechanisms, including the stimulation of cell proliferation, migration, differentiation, survival, DNA repair and the induction of enzymes involved in the detoxification of reactive oxygen species [20]. Subsequent pre-clinical studies have shown that palifermin has a remarkable ability to protect many different epithelial tissues from a variety of toxic insults.

Pre-clinical studies have explored multiple areas of application. The largest effort has focused on palliative care in the oncology setting, as it was postulated that palifermin could protect tissues from the harmful effects of chemoradiotherapy regimens, thus limiting the toxicities associated with their use, such as mucositis, xerostomia and diarrhoea. The advantages of pursuing such a course were obvious as the timing of palifermin treatment relative to the toxic exposure could be readily controlled. This was particularly appropriate as the beneficial effects of palifermin typically are most pronounced when it is administered prior to the noxious agent. Other studies have tested the effects of palifermin in the context of acute organ injury such as ALI. Clearly, in these applications, palifermin cannot be administered before injury occurs, and so the strategy is dependent upon the ability of palifermin to limit the extent of tissue damage after injury and/or to accelerate repair processes. Pre-clinical studies suggest that palifermin might have utility as a biodefense therapeutic for public and/or emergency personnel to mitigate radiation syndromes in situations when radioactive material has been released.

In some cases, pre-clinical studies have provided important new insights into KGF function, and in doing so have suggested new avenues of investigation to be pursued. For example, it was originally proposed that palifermin might limit GVHD by reducing injury to the GI tract that results from chemoradiotherapy conditioning regimens prior to BMT. While several BMT models demonstrated a beneficial role for palifermin in reducing GVHD, subsequent studies indicated an immunomodulatory mechanism of palifermin that is in addition to, and independent of, its ability to limit the damage to epithelial tissues caused by conditioning regimens.

The accepted paradigm of KGF functioning as a mesenchymal mediator of epithelial growth and regeneration was challenged by the observations that KGF family members act as pre-synaptic organizing molecules. The differential formation of excitatory (glutamate-mediated) and inhibitory (GABA-mediated) synapses is critical for normal brain function, and an imbalance of these synapses may play a role in the pathogenesis of various neurological disorders associated with abnormal synapse formation such as epilepsy, autism, schizophrenia and Tourette's syndrome. These studies demonstrate an unexpected new area for potential clinical development of palifermin.

The results described in this review document the remarkable ability of palifermin to protect or facilitate the repair of many different epithelial tissues following injury in pre-clinical models. The goal of such studies was to provide supporting evidence for efficacy in corresponding clinical situations. The accompanying article in this series reviews current information about palifermin use in a number of clinical trials primarily involving palliative care in the cancer setting. While the findings of some clinical trials have borne out the positive results from pre-clinical studies, others have been less convincing (see accompanying review article in this series). Such inconsistencies between pre-clinical models and clinical studies probably reflect inherent differences between animal and human biology, as well as sub-optimal dosing regimens that will require further adjustment. Furthermore, palifermin might be used in combination with other pharmaceutical agents in a particular setting to maximize its beneficial effects. Nonetheless, animal models have been invaluable in demonstrating the pharmaceutical potential of palifermin, and will likely continue to be of considerable importance in promoting further clinical development.
